# The psychological resilience of teenagers in terms of their everyday emotional balance and the impact of emotion regulation strategies

**DOI:** 10.3389/fpsyg.2024.1381239

**Published:** 2025-02-20

**Authors:** Zengyan Yu, Wen Liu

**Affiliations:** ^1^School of Mental Health, Qiqihar Medical University, Qiqihar, China; ^2^School of Psychology, Liaoning Normal University, Dalian, China

**Keywords:** adolescents, daily stress, psychological resilience, emotion regulation strategies, emotional balance

## Abstract

**Objective:**

Adolescents are also more vulnerable to the effects of everyday life stimuli and exhibit a range of negative emotional states that can develop into severe affective disorders. However, Psychological resilience maybe enable the prevention of emotional problems associated with daily stress rather than intervening treating the problem after it has occurred.

**Methods:**

A total of 104 individuals (54 participants in the high psychological resilience group and 50 participants in the low psychological resilience group) were first identified. Then, the 8-day experiential sampling method was used to determine the characteristics of adolescents with different psychological resilience levels in terms of emotional balance under daily stress. Further combined with diary method research, a multilayered linear model was used to explore the predictive effects of six emotion regulation strategies on adolescents’ emotional balance.

**Results:**

The obtained Results show that high psychological resilience adolescents demonstrated higher levels of emotional balance and positive rates and lower rates of change in emotional balance than low psychological resilience adolescents. In terms of facilitating emotion regulation strategies, high psychological resilience was associated with greater use of cognitive reappraisal and social sharing strategies (which positively predicted emotional balance under daily stress) and less use of expression suppression and rumination strategies (which negatively predicted levels of emotional balance).

**Conclusion:**

Adolescents with high psychological resilience exhibit good adaptive emotional states in daily stressful situations, which is closely related to their use of adaptive emotion regulation strategies such as cognitive reappraisal and social sharing and may be useful for further intervention research.

## 1 Introduction

Adolescents are under pressure from school, family and society in a highly competitive society and face unbalanced physical and mental development, leading to a greater probability of psychopathology in this age group. According to data released by the National Health Commission in 2018, approximately 30 million children and adolescents younger than 17 years in China suffer from various emotional disorders or problems. The emotional problems of adolescents are more prominent due to the influence of physiological and social factors. Emotional health problems in daily life, however, depend on the outcome of positive and negative emotional processing—emotional balance, which is a comprehensive expression of adaptive emotional experience under daily stress and can significantly impact individuals’ emotional health (Veilleux et al., 2020). However, there are few studies on the comprehensive characteristics of emotional balance at this stage, and the ecological validity of the current mainstream research methods (such as questionnaires and experimental methods) is low. In recent years, experience sampling methods for transiently assessing daily positive and negative emotions have gradually attracted increasing amounts of attention. Currently, the experience sampling method has two main versions: one measures emotional states multiple times at equal intervals throughout the day and for several days in a row (Brans et al., 2013; Lyu et al., 2017); the other measures emotional states once a day, mainly at the end of the day, similar to diary studies (Kalokerinos et al., 2017; Troy et al., 2019). Domestic and international scholars, using the experience sampling method, have focused mainly on the daily emotional characteristics of adults and have given little consideration to their influencing factors. The brain is highly plastic in the early stages of adolescence, making adolescents vulnerable to both the negative effects of stress/adversity and making the period critical for appropriate prevention or intervention (Malhi et al., 2019). Therefore, appropriate measures need to be taken to promote and maintain emotional balance under daily stress in early adolescents, thereby benefiting their healthy physical and mental development.

### 1.1 Theoretical basis of the study

Psychological resilience research aims to identify effective strategies for preventing and mitigating emotional problems under stress/adversity and promoting the emotional well-being of individuals (Yang, 2020). The dual processing theory of psychological resilience provides a theoretical basis for exploring the mechanisms of psychological resilience (Tugade, 2011). Psychological resilience involves two types of information processing: positive and negative emotion processing, which focuses on the outcome of daily emotional balance; and controlled and automatic processing, which focuses on the interplay between external and internal emotion regulation. Dual processing theory emphasizes the mechanism of psychological resilience as a process in which different types of emotion regulation interact with everyday emotions. In addition, recent research has proposed a framework for psychological resilience impact regulation whereby a family of emotion regulation strategies predicts short-term consequences such as emotions and thus psychological resilience, emphasizing the need for the two main psychological resilience methods, stress and coping methods, in addition to emotions and emotion regulation, to be organically integrated to complement each other’s strengths (Troy et al., 2023).

### 1.2 Current status and aim

However, a few issues still need to be further explored. First, psychological resilience under everyday stress conditions is often overlooked. In contrast to the stress resilience that arises in the presence of severe adversity, psychological resilience enables the prevention of emotional problems associated with everyday stress rather than intervening in treating the problem after it has occurred (Bonanno and Diminich, 2013; Kalisch et al., 2017). Therefore, it is important and even necessary to explore the impact of psychological resilience on adolescents’ emotional balance under stressful daily conditions to not only bridge the gap in traditional pathophysiological research but also improve adolescents’ adaptive emotional experiences under stressful daily conditions. However, it is challenging to shape and guide psychological resilience research to practically enrich the field of emotional health science and improve its level of application in clinical practice. Second, previous studies have yielded inconsistent findings, e.g., regarding whether highly resilient individuals have higher levels of positive emotions (Block and Kremen, 1996). Moreover, findings are inconsistent as to whether they also exhibit lower levels of negative emotions, and there is less research on the level of emotional balance and other related characteristics that are the result of processing positive and negative emotions. Third, previous studies have used measures of emotions and emotion regulation strategies with low methodological ecological validity.

The present study was a person-centered study conducted during the summer through the experience sampling method. The aim of this study was to explore the emotional balance characteristics of adolescents with different levels of psychological resilience. We subsequently explored the role of emotion regulation strategies as an important psychological resilience resource for individuals in promoting daily emotional balance, which has certain practical significance for improving adolescents’ adaptive emotional responses under daily stress and promoting their emotional health.

## 2 Materials and methods

### 2.1 Participants

Convenience sampling was used to select 616 junior high school students aged 7–8 years from a middle school in China for questionnaire surveys; 534 valid questionnaires were used to test their psychological resilience through a variety of methods and group validity testing. Additionally, 104 people were ultimately identified to participate in the experience sampling study (54 in the high psychological resilience group and 50 in the low psychological resilience group), with an age range of 12–15 years and a mean age of 13.15 ± 0.81 years.

### 2.2 Instruments

#### 2.2.1 Daily emotional experience record sheet

The scale was developed and revised by Wei. The revised version of the scale includes 10 items each for positive and negative emotional descriptors, and participants were asked to recall their emotional states in the past 2 h on a five-point scale (Wei et al., 2017).

#### 2.2.2 Adolescent life events scale

The scale was developed for secondary school students and asks participants to indicate whether the events on the scale had occurred and about their degree of influence in the past 12 months; this scale includes a total of five factors and 26 items (Xin and Yao, 2015). The internal consistency reliability of the scale was 0.91.

#### 2.2.3 Psychological resilience scale

The Connor-Davidson-10 by Campbell-Sills and Stein (2007) was used; it has 10 items with high reliability and validity. The internal consistency reliability of the scale was 0.83.

#### 2.2.4 Adolescent psychological resilience scale

The scale measures psychological resilience in adolescents and was developed based on traditional Chinese culture. The scale consists of 27 items and contains two factors (Hu and Gan, 2008).

#### 2.2.5 Subjective happiness index scale

This scale contains nine items, eight of which constitute the overall affective index, one of which is the life satisfaction index, and the weighted sum of the two is the overall happiness index (Wang et al., 1999). The internal consistency reliability of the scale was 0.872.

#### 2.2.6 Questionnaire on the use of daily emotion regulation strategies

Adapted from previous studies (Brans et al., 2013; Kalokerinos et al., 2017; Troy et al., 2019), this questionnaire examines participants’ use of the six emotion regulation strategies under daily stress and their effects on emotional experience.

### 2.3 Procedure

#### 2.3.1 Subject grouping

The specific high and low group screening methods were divided into three steps: (1) potential profile analysis of all question items was performed according to the psychological resilience scale to determine the dividing line between high and low categories; (2) considering the impact of stress on psychological resilience, the variable-centered convergence operation method was used for further screening; and (3) the above two methods to determine the final candidate for the participants and the Adolescent Psychological Resilience Scale were combined to test the validity of the grouping (see Supplementary material).

#### 2.3.2 Measurement of daily emotional balance

First, the requirements of the experience sampling survey were explained to the participants; then, the measurements were taken three times a day for eight consecutive days (see Supplementary material).

#### 2.3.3 Measurement of daily emotion regulation strategies

Every day before the end of school, a daily emotion regulation strategy use questionnaire was issued to each subject. The participants were asked to complete the survey before going to bed, similar to a diary. A person was assigned to each class to collect and organize the data the next morning for a total of 8 consecutive days of measurement.

### 2.4 Statistical methods

The data were organized and analyzed by SPSS 22.0, Mplus 7.4, and Mintab 19.0. Emotional intensity is the subjective intensity of an individual’s reported emotional experience, and emotional frequency is how often an individual experiences a certain emotion over a period (Lyu et al., 2017). Emotional balance is the difference between positive and negative emotional scores; emotional instability was calculated to reflect the general rate of change in positive and negative emotions through the mean square successive difference (MSSD), which can be automatically calculated using Mintab 19.0 software. A total of 2,060 valid data points were collected over the 7 days of experiential sampling. The average valid response rate was 96.17%, of which the highest number of valid data points was 21 and the lowest was 12. There was no significant difference in the effective response rate between the participants in the high and low psychological resilience groups, *t*(100) = 1.61, *p* = 0.11.

In addition, multilevel linear modeling analysis was conducted with HLM6 software, and the model of this study included two levels, with the number of times or days nested at the individual level, meaning that the first level was the intraindividual level, including time and the degree of use of the six emotion regulation strategies, and the second inter-individual level included the variables of sex, age, and group; the dependent variable was the number of positive and negative emotional experiences after 1–2 h. Multilevel modeling analysis was used to explore the predictive effects of different emotion regulation strategies on daily emotional experience (see Supplementary material).

## 3 Results

### 3.1 Characteristics of the daily emotional balance of adolescents across psychological resilience levels

In terms of the indicators of emotional balance and positive rate, adolescents with high psychological resilience had higher mean scores and positive rates of emotional balance; in terms of the rate of change in emotional balance, there was no significant difference between adolescents with different psychological resilience levels in terms of the positive emotional general rate of change, but the general rate of change in negative emotion and emotional balance was significantly higher in the lower psychological resilience group than in the higher psychological resilience group (see Table 1).

**Table 1:** Comparison of the emotional balance of youth across psychological resilience levels.

Emotion balance characteristics	High resilience group (*n* = 54)	Low resilience (*n* = 47)	*t*	Cohen’s *d*
Positive emotion mean score	36.39 ± 6.08	26.50 ± 6.16	8.15***	1.62
Positive emotional intensity	3.76 ± 0.54	2.95 ± 0.48	8.00***	1.59
Positive emotion frequency (%)	91.11 ± 15.44	82.96 ± 16.73	2.56*	0.51
Negative emotions mean Score	15.72 ± 5.04	21.12 ± 7.11	−4.46***	−0.88
Negative mood intensity	2.40 ± 0.33	2.79 ± 0.53	−4.53***	−0.89
Negative mood frequency (%)	38.79 ± 28.48	51.94 ± 26.47	−2.41*	−0.48
Average emotional balance	20.67 ± 8.55	5.38 ± 10.83	7.95***	1.57
Average positive rate	2.64 ± 0.81	1.56 ± 0.65	7.38***	1.47
Positive emotion general change rate	24.13 ± 20.93	29.38 ± 20.02	−1.29	−0.26
Negative emotion general change rate	9.20 ± 10.42	22.69 ± 19.07	−4.50***	−0.88
Emotional balance general change rate	45.05 ± 42.94	75.37 ± 54.73	−3.13**	−0.62

*p < 0.05, **p < 0.01, ***p < 0.001.

### 3.2 Effects of different emotion regulation strategies on emotional balance in teenagers

#### 3.2.1 Test for inter-individual differences in emotional balance among adolescents

A null model (unconditional model) was first developed through Model 1 to test whether there were inter-individual differences in emotional balance and both dimensions under daily stress. The ICC was calculated and reported to determine whether a multilevel linear model was necessary. In Model 1, fixed-part parameter estimates indicated that positive emotions, negative emotions, and emotional balance under daily stress were significantly different between individuals (*γ_00_* = 15.02, *SE* = 0.51, *t* = 29.35, *p* < 0.001; *γ_00_* = 13.37, *SE* = 0.45, *t* = 29.53, *p* < 0.001; *γ_00_* = 1.65, *SE* = 0.83, *t* = 1.99, *p* < 0.05). As seen from the parameter estimates in the randomized section, there was also significant inter-individual variability in positive emotions, negative emotions, and emotional balance under daily stress (*τ*_00_ = 23.23, *χ*^2^ = 838.92, *df* = 100, *p* < 0.001; *τ*_00_ = 18.63, *χ*^2^ = 1028.64, *df* = 100, *p* < 0.001; *τ*_00_ = 61.72, *χ*^2^ = 951.12, *df* = 100, *p* < 0.001). The proportions of variation between groups and the total variation were 49.79, 55.32, and 53.30%, respectively, which were greater than 5%, indicating the need for a mixed model.

#### 3.2.2 Predictive effects of different emotion regulation strategies on emotional balance under daily stress

Model 2 is a random coefficient regression model with six emotion regulation strategies added at the first level. The model is mainly used to test the predictive effects of emotion regulation strategies on positive emotions, negative emotions, and emotional balance. The results of Model 2 indicated that, according to the fixed effects analysis (Table 2), under daily stress, both the cognitive reappraisal strategy and the expressive inhibition strategy were strongly associated with an increase in positive emotional experiences and a decrease in negative emotional experiences (*p* < 0.05); the rumination strategy was strongly associated with an increase in negative emotional experiences (*p* < 0.001); and the social sharing strategy was strongly associated with a decrease in negative emotional experiences (*p* < 0.05).

**Table 2:** Effects of emotion regulation strategies on emotional balance.

Parameter	Fixed effect					
	Positive emotions		Negative emotions		Emotional balance	
	*B (SE)*	*p*	*B (SE)*	*p*	*B (SE)*	*p*
Intercept (γ_00_)	14.93 (0.44)	<0.001	13.32 (0.35)	<0.001	1.59 (0.65)	0.02
Distraction (γ_10_)	0.13 (0.20)	0.52	0.27 (0.15)	0.07	−0.11 (0.31)	0.73
Rumination (γ_20_)	−0.34 (0.24)	0.15	0.66 (0.17)	<0.001	**−1.09 (0.34)**	<0.01
Cognitive reappraisal (γ_30_)	0.54 (0.22)	0.02	−0.33 (0.17)	<0.05	**0.70 (0.35)**	<0.05
Expressive inhibition (γ_40_)	−0.53 (0.20)	0.01	0.76 (0.15)	<0.001	**−1.34 (0.31)**	<0.001
Acceptance strategy (γ_50_)	0.13 (0.24)	0.60	0.03 (0.22)	0.89	−0.03 (0.38)	0.93
Social sharing (γ_60_)	0.34 (0.23)	0.15	−0.34 (0.15)	0.02	**0.70 (0.33)**	0.04

Bolded portions indicate effect size values for the four emotion regulation strategies that were significant predictors of emotional balance.

Randomized partial parameter estimates indicated significant random effects for the expression suppression and social sharing strategies when predicting positive emotions; i.e., there were significant interindividual differences in the effects of these two emotion regulation strategies on the experience of positive emotions. When predicting emotional balance, the random effect of the rumination strategy was significant (*p* < 0.05), indicating that there were significant inter-individual differences in the effect of the rumination strategy on emotional balance.

#### 3.2.3 Moderating effects test of the relationship between emotion regulation strategies and emotional balance

Model 3 is a randomized coefficient regression model with the predictor variables sex, age, and group at the second level; this model is designed to explore whether sex, age, and group moderate the relationship between daily emotion regulation strategies and emotional balance.

First, in terms of cognitive reappraisal strategies, age significantly moderates the relationship between cognitive reappraisal and negative emotions (*γ_32_* = 0.44, *SE* = 0.20, *t* = 2.19, *p* < 0.05), and age significantly moderates the relationship between cognitive reappraisal and positive emotions (*γ_32_* = −0.52, *SE* = 0.23, *t* = −2.24, *p* < 0.05).

Sex and age significantly moderate the relationship between distraction strategy and positive mood (*γ_11_* = −1.10, *SE* = 0.38, *t* = −2.89, *p* < 0.01; *γ_12_* = 0.54, *SE* = 0.21, *t* = 2.63, *p* < 0.05), and sex and age significantly moderate the relationship between distraction strategies and emotional balance (*γ_11_* = −1.82, *SE* = 0.59, *t* = −3.07, *p* < 0.01; *γ_12_* = 0.80, *SE* = 0.35, *t* = 2.29, *p* < 0.05). Group moderately affects the relationship between distraction strategies and negative emotional experiences (*γ_13_* = −0.60, *SE* = 0.29, *t* = −2.08, *p* < 0.05).

Group also moderates the relationship between the expression inhibition strategy and negative emotions (*γ_43_* = −0.72, *SE* = 0.29, *t* = −2.49, *p* < 0.05) and the relationship between the expression inhibition strategy and emotional balance (*γ_43_* = 1.54, *SE* = 0.55, *t* = 2.82, *p* < 0.01).

## 4 Discussion

### 4.1 Comparison of the emotional balance characteristics of adolescents with different levels of psychological resilience

First, in terms of the dimensions of positive and negative emotions, there were differences in the intensity and frequency of positive and negative emotional experiences under daily stress among middle school students with different levels of psychological resilience. In addition, the present study demonstrated that highly psychologically resilient adolescents also experienced fewer negative emotions, which is not entirely consistent with the results of previous studies. For example, while a study revealed that high school students with high psychological resilience had relatively low negative emotions (Xi et al., 2013), other researchers have found no significant differences in negative emotions among college students with different levels of psychological resilience (Tugade and Fredrickson, 2004); moreover, a study with older adults as participants even found that individuals with high psychological resilience experienced more negative emotions (Ong et al., 2006). The inconsistent conclusions regarding the relationship between psychological resilience and negative emotions may be influenced by age, stress intensity, and other factors; these conclusions should be further explored and verified through systematic evaluation and meta-analysis in the future.

Second, in terms of the level of emotional balance, the results of this study indicate that adolescents with high psychological resilience have greater balance, which is consistent with the findings of previous studies (Fredrickson, 2010; Lyu et al., 2017); additionally, emotional balance serves as an important component of individuals’ subjective sense of well-being (Diener et al., 2010), and greater emotional balance may be associated with lower depression and other mood disorders (Sumi, 2014). The present study also revealed that highly psychologically resilient adolescents exhibited a high rate of positivity (2.64:1) but did not meet Fredrickson’s (2010) criterion of a minimum rate of positivity for thriving (3:1), which may be closely related to an increase in negative emotional experiences with age at this stage of life. Adolescents face a wide range of physiological, social, and psychological changes, and the prefrontal cortex, which is responsible for self-control and decision-making, has not yet been fully developed and is at an incomplete stage of emotional and cognitive maturity. They cannot yet control and regulate their strong negative emotional experiences in the face of academic and interpersonal pressures in daily life. However, relatively few studies on the rates of positivity exist, and it is necessary to further explore whether positivity is affected by age, sex, geographic location, or even psychological flexibility.

Finally, in terms of emotional balance and the temporal dynamic properties of the two dimensions, the results suggest that under daily stress, individuals with low psychological resilience have higher rates of change in negative emotions in general and in emotional balance, a characteristic that is not beneficial to their physical and mental health. As studies have shown that greater variability and instability in both positive and negative emotions are associated with poorer mental health (Gruber et al., 2013; Gilboa and Nahum, 2022) and that this effect is more prominent for individuals with negative emotions than for those with positive emotions (Houben et al., 2015), it is evident that instability of emotional dynamic patterns is a predictor of increased risk of psychiatric disorders when experiencing everyday stress.

### 4.2 Impact of various emotion regulation strategies on adolescents’ emotional balance

#### 4.2.1 Effects of different emotion regulation strategies

This study showed that different daily emotion regulation strategies do not play the same role. First, the cognitive reappraisal strategy was strongly associated not only with an increase in positive emotions and emotional balance but also with a decrease in negative emotional experiences. This result is consistent with previous findings. The cognitive reappraisal strategy is the most researched and effective emotion regulation strategy. This strategy is positively correlated with psychological resilience and can effectively regulate individuals’ negative emotions (Troy et al., 2013). However, this strategy is affected by the duration of stress, which may lead to more effective emotion regulation strategies under everyday stress and less effectiveness in solving emotional reactivity or prolonged emotional problems (Webb et al., 2012); moreover, expression suppression is negatively related to psychological resilience, reduces positive emotions and increases negative emotional experiences during everyday stress. However, it has also been shown that expressive inhibition can be situationally influenced and that whether the inhibitory content is informational or affective can have dramatically different effects on individuals (Polizzi and Lynn, 2021). Additionally, rumination strategies may prevent individuals from extricating themselves from everyday stressors, thus leading to negative emotional experiences and perceptions, which, at least in the short term, can lead to the maintenance and increase of negative emotions (Volkaert et al., 2020) and a decrease in positive emotional experiences (Polizzi and Lynn, 2021), which is strongly positively associated with expression inhibition strategies (Johnson et al., 2016). However, social sharing strategies were related to adaptations of emotion regulation strategies in this study, which are strongly associated with an increase in positive emotional experiences under daily stress, but social sharing strategies were used less frequently in the adolescent stage, which may be related to the characteristics of psycho-cognitive development in adolescence.

However, this study did not find a predictive effect of the distraction strategy or acceptance strategy on emotional balance or either dimension under daily stress. For this reason, through moderated effects analysis, the present study revealed that the relationship between distraction strategy and daily emotional experience under daily stress is affected by age, sex, and group, which is inconsistent with the results of previous related studies showing an association between distraction strategy and an increase in positive emotions in adults (Brans et al., 2013); however, some studies have shown that a distraction strategy stably positively predicts negative emotions (Troy et al., 2019). The distraction strategy is not a stable and effective emotion regulation strategy under daily stress and can be affected by several factors. In contrast, the acceptance strategy, similar to the core concept of allowing nature to take its course in positivity, is positively associated with psychological resilience when coping with daily stress (Polizzi and Lynn, 2021); however, in the present study, this strategy was not strongly associated with self-reported experience of cumulative emotions, and research suggests that the strategy may be more effective for reducing only immediate physiological responses following stressful stimuli (Troy et al., 2019).

#### 4.2.2 Role and application of emotion regulation strategies from a psychological resilience perspective

Cognitive emotion regulation strategies may be an important cognitive resource for individuals with high psychological resilience and may significantly impact their emotional well-being (Powers et al., 2015; Malhi et al., 2019). The results of the present study also suggest that cognitive reappraisal and sharing strategies are important cognitive resources for psychological resilience in adolescents. These strategies include manifesting adaptive emotion regulation strategies under daily stress, maintaining and enhancing positive emotional experiences, decreasing negative emotional experiences, decreasing physiological stress levels, and ultimately contributing to daily emotional equilibrium, whereas expression inhibition and rumination manifest as maladaptive emotion regulation strategies under daily stress; these strategies are more likely to generate and increase negative emotional experiences and reduce emotional homeostasis and may also chronically elicit increased levels of amygdala and sympathetic activation. Chronically higher physiological levels of arousal are detrimental to an individual’s physical and psychological well-being (Ochsner et al., 2004). In conclusion, adolescents with high psychological resilience may be more likely to use positive appraisal strategies such as cognitive reappraisal, less likely to use over-focused negative appraisal strategies such as rumination strategies on the emotional input side, more likely to use positive resolution strategies such as social sharing, and less likely to use negative coping strategies such as expressive inhibition on the emotional output side, thereby promoting emotional balance under daily stress. This finding is largely in line with a positive appraisal style promoting emotional balance in adolescents under daily stress through positive appraisal patterns such as positive valuing and coping, and this positive bias model is the underlying mechanism of action in psychopathology with broader implications (Kalisch et al., 2015).

From an intervention application perspective, highly psychologically resilient individuals may implement effective emotion regulation strategies before, during, and after stress/adversity to mitigate the negative effects of stress (Lee et al., 2019). In both clinical and non-clinical samples, the relationship between psychological resilience and emotional well-being may be strengthened through training in emotion regulation strategies; i.e., the development of emotion regulation skills attenuates the arousal of emotions by adversity/stress, thereby protecting individuals from psychopathology (Polizzi and Lynn, 2021). How highly resilient individuals maintain and increase their positive emotional experiences through adaptive emotion regulation strategies is a key component in explaining the mechanisms underlying the phenomenon of psychological resilience (Hopp et al., 2011). When an individual is in a stressful situation, appropriate emotion regulation strategies help him or her maintain more positive and calming emotional responses and thus more effective psychological resilience. They thus cope with the stressors more effectively. In the future, we need to explore the facilitation of implicit emotion regulation and the predictive effects of emotion regulation on the composite profile of emotional balance.

## Data availability statement

The raw data supporting the conclusions of this article will be made available by the authors, without undue reservation.

## Ethics statement

The studies involving humans were approved by Project Review Report of the Ethics Committee of Qiqihar Medical College ([2021] No. 123) Project Title: Study on the Characteristics, Influencing Factors and Promotion of youth’ Daily Emotions from the Perspective of Psychological Resilience. This study did have the consent and support of the school where the subjects were enrolled. The studies were conducted in accordance with the local legislation and institutional requirements. The participants provided their written informed consent to participate in this study.

## Author contributions

ZY: Data curation, Funding acquisition, Methodology, Writing – original draft. WL: Conceptualization, Writing – review & editing.
