# Supplementary material for: The psychological resilience of teenagers in terms of their everyday emotional balance and the impact of emotion regulation strategies
Source: Front Psychol. 2025 Feb 20;15:1381239. doi: 10.3389/fpsyg.2024.1381239 (PMC11883688; doi:10.3389/fpsyg.2024.1381239)
Supplement: Supplementary file 1 [file Presentation_1.pdf]

Supplementary material

1. Approach to psychological resilience grouping

After potential profile analysis, 534 adolescents were potentially profiled based on the Psychological Resilience Scale question items, and 1-5 potential categories were extracted. The results for each indicator are shown in Table S1. The entropy indicator was 0.80 when the number of categories was 2, indicating that the representative classification accuracy was more than 90% (Hu et al., 2017). The results of the LMRT also showed that the 2-category model was better than the 1-category model, p<0.001, and the improvement in optimization obtained by indicators such as the AIC, BIC, and ssaBIC varied less with increasing category number in the model after category number 2. Although the difference between the category 3 and 4 models is significant at the 0.05 level, their entropy metrics begin to decline. Therefore, the 2-category model was deemed to describe the type information more adequately; the final numbers of high and low groups were determined to be 275 and 268, respectively; and the attribution probability matrices of the two potential categories were 0.93 and 0.96, respectively.

The convergence operation method First, the total stress scores of all the adolescents were ranked in order of high to low stress. According to the criteria of previous research (Lv Mengshi et al., 2017), the top 50% of the participants were considered "potentially psychologically resilient". A total of 267 participants (128 males and 139 females) fulfilled the preconditions for the definition of psychological resilience. The participants were further ranked according to their subjective well-being from high to low scores, in which the initial selection criteria for the high psychological resilience group were 27% of the top scores in terms of subjective well-being, totaling 60 participants, and the low psychological resilience group was 27% of the bottom scores in terms of subjective well-being, totaling 56 participants.

Group validity test Comprehensive convergence of the results of the operational method and potential profile analysis were tested. Among the 54 participants in the high psychological resilience group and 50 participants in the low psychological resilience group, three participants did not participate in the survey for three consecutive days. Accordingly, these data were invalidated, and ultimately, 101 participants were valid, 54 in the high psychological resilience group; and 47 in the low psychological resilience group. The validity of screening the high and low groups was then verified using the Adolescent Psychological Resilience Scale, $t = 26.25$, $p < 0.001$. Different levels of psychological resilience can be differentiated by combining person-centered potential profiling and variable-centered convergent operations and group validity tests, which can be combined to screen for greater validity through mutual testing.

2. Measurement of daily emotional balance

Three time points were selected from 9:00 am to 3:00 pm, with an interval of 2-3 hours between each pair of sampling points. A paper questionnaire was used for group measurements three times a day at a fixed location and took 5-10 minutes to complete, to minimize disruption of the school's work schedule. However, because the data from the three sessions on the first day were used as an exercise, the data from the seven days of sampling were statistically analyzed.

3. Statistical methods

Methods of calculating emotion intensity and frequency In rating the PANAS-C scale, the 1-point option indicates that the participant "hardly ever" experienced the given emotion at the moment. Therefore, the intensity of the emotion was calculated by summing the average of all ratings greater than 1 corresponding to those descriptors as the intensity of the emotion, and the proportion of ratings greater than 1 corresponding to the number of valid samples was calculated as the frequency of the emotion. The intensity and frequency of each of the 10 positive and 10 negative emotions were then averaged for each adolescent.

The frequency of daily emotion regulation strategy use was calculated in the same way as emotion intensity and frequency, by summing and averaging all validly sampled ratings of that subject for which the frequency of that emotion regulation strategy was greater than 1 point (with 1 point indicating little to no use), and was used as the subject's score for intensity of use for that emotion regulation strategy. Alternatively, specific emotion regulation strategy ratings of 1 were scored 0, and rating of 2-5 were scored 1. The proportion of each emotion regulation strategy frequency to effective sampling was then counted as a score for the frequency of use of that emotion regulation strategy (Troy et al., 2019).

4. Random effects modeling equation via HLM6

Model1 Level-1 $\text{Affect}_{tj} = \beta_{0j} + r_{ij}$

Level-2 $\beta_{0j} = \gamma_{00} + u_{0j}$

Model2

Level-1 Within-Individual Level

$\text{Affect}_{tj} = \beta_{0j} + \beta_{1j}$ (distraction strategy) + β_{2j} (rumination strategy) + β_{3j} (cognitive reappraisal) + β_{4j} (expression suppression) + β_{5j} (acceptance strategy) + β_{6j} (social sharing) + r_{ij}

Level-2 Inter-individual Level

$$\beta_{0j} = \gamma_{00} + u_{0j}$$

$$\beta_{1j} = \gamma_{10} + u_{1j}$$

$$\beta_{2j} = \gamma_{20} + u_{2j}$$

$$\beta_{3j} = \gamma_{30} + u_{3j}$$

$$\beta_{4j} = \gamma_{40} + u_{4j}$$

$$\beta_{5j} = \gamma_{50} + u_{5j}$$

$$\beta_{6j} = \gamma_{60} + u_{6j}$$

Model3

Level-1 Within-Individual Level

Affect$_{tj}$ = β_{0j} + β_{1j} (distraction strategy) + β_{2j} (rumination strategy) + β_{3j} (cognitive reappraisal) + β_{4j} (expression suppression) + β_{5j} (acceptance strategy) + β_{6j} (social sharing) + r_{ij}

Level-2 Inter-individual Level

$$\beta_{0j} = \gamma_{00} + \gamma_{01}(\text{gender}_j) + \gamma_{02}(\text{age}_j) + \gamma_{03}(\text{group}_j) + u_{0j}$$

$$\beta_{1j} = \gamma_{10} + \gamma_{11}(\text{gender}_j) + \gamma_{12}(\text{age}_j) + \gamma_{13}(\text{group}_j) + u_{1j}$$

$$\beta_{2j} = \gamma_{20} + \gamma_{21}(\text{gender}_j) + \gamma_{22}(\text{age}_j) + \gamma_{33}(\text{group}_j) + u_{2j}$$

$$\beta_{3j} = \gamma_{30} + \gamma_{31}(\text{gender}_j) + \gamma_{32}(\text{age}_j) + \gamma_{33}(\text{group}_j) + u_{3j}$$

$$\beta_{4j} = \gamma_{40} + \gamma_{41}(\text{gender}_j) + \gamma_{42}(\text{age}_j) + \gamma_{43}(\text{group}_j) + u_{4j}$$

$$\beta_{5j} = \gamma_{50} + \gamma_{51}(\text{gender}_j) + \gamma_{52}(\text{age}_j) + \gamma_{53}(\text{group}_j) + u_{5j}$$

$$\beta_{6j} = \gamma_{60} + \gamma_{61}(\text{gender}_j) + \gamma_{62}(\text{age}_j) + \gamma_{63}(\text{group}_j) + u_{6j}$$
